# Serum glial fibrillary acidic protein in natalizumab-treated relapsing-remitting multiple sclerosis: An alternative to neurofilament light

**DOI:** 10.1177/13524585231188625

**Published:** 2023-08-02

**Authors:** Mark HJ Wessels, Zoë YGJ Van Lierop, Samantha Noteboom, Eva MM Strijbis, Johannes A Heijst, Zoé LE Van Kempen, Bastiaan Moraal, Frederik Barkhof, Bernard MJ Uitdehaag, Menno M Schoonheim, Joep Killestein, Charlotte E Teunissen

**Affiliations:** Department of Neurology, MS Center Amsterdam, Amsterdam Neuroscience, Amsterdam UMC, location VUmc, Vrije Universiteit Amsterdam, Amsterdam, The Netherlands; Department of Anatomy and Neurosciences, MS Center Amsterdam, Amsterdam Neuroscience, Amsterdam UMC, location VUmc, Vrije Universiteit Amsterdam, Amsterdam, The Netherlands; Department of Neurology, MS Center Amsterdam, Amsterdam Neuroscience, Amsterdam UMC, location VUmc, Vrije Universiteit Amsterdam, Amsterdam, The Netherlands; Department of Clinical Chemistry, MS Center Amsterdam, Amsterdam Neuroscience, Amsterdam UMC, location VUmc, Vrije Universiteit Amsterdam, Amsterdam, The Netherlands; Department of Neurology, MS Center Amsterdam, Amsterdam Neuroscience, Amsterdam UMC, location VUmc, Vrije Universiteit Amsterdam, Amsterdam, The Netherlands; Department of Radiology and Nuclear Medicine, MS Center Amsterdam, Amsterdam Neuroscience, Amsterdam UMC, location VUmc, Vrije Universiteit Amsterdam, Amsterdam, The Netherlands; Department of Radiology and Nuclear Medicine, MS Center Amsterdam, Amsterdam Neuroscience, Amsterdam UMC, location VUmc, Vrije Universiteit Amsterdam, Amsterdam, The Netherlands; Queen Square Institute of Neurology, Centre for Medical Image Computing, University College London, London, UK; Department of Neurology, MS Center Amsterdam, Amsterdam Neuroscience, Amsterdam UMC, location VUmc, Vrije Universiteit Amsterdam, Amsterdam, The Netherlands; Department of Anatomy and Neurosciences, MS Center Amsterdam, Amsterdam Neuroscience, Amsterdam UMC, location VUmc, Vrije Universiteit Amsterdam, Amsterdam, The Netherlands; Department of Neurology, MS Center Amsterdam, Amsterdam Neuroscience, Amsterdam UMC, location VUmc, Vrije Universiteit Amsterdam, Amsterdam, The Netherlands; Department of Clinical Chemistry, MS Center Amsterdam, Amsterdam Neuroscience, Amsterdam UMC, location VUmc, Vrije Universiteit Amsterdam, Amsterdam, The Netherlands

**Keywords:** Multiple scleroris, natalizumab, glial-fibrillary-acidic-protein, neurofilament light, disability-progression, MRI volumetrics

## Abstract

**Background:**

There is a need in Relapsing-Remitting Multiple Sclerosis (RRMS) treatment for biomarkers that monitor neuroinflammation, neurodegeneration, treatment response, and disease progression despite treatment.

**Objective:**

To assess the value of serum glial fibrillary acidic protein (sGFAP) as a biomarker for clinical disease progression and brain volume measurements in natalizumab-treated RRMS patients.

**Methods:**

sGFAP and neurofilament light (sNfL) were measured in an observational cohort of natalizumab-treated RRMS patients at baseline, +3, +12, and +24 months and at the last sample follow-up (median 5.17 years). sGFAP was compared between significant clinical progressors and non-progressors and related to magnetic resonance imaging (MRI)-derived volumes of the whole brain, ventricle, thalamus, and lesion. The relationship between sGFAP and sNfL was assessed.

**Results:**

A total of 88 patients were included, and 47.7% progressed. sGFAP levels at baseline were higher in patients with gadolinium enhancement (1.3-fold difference, *p* = 0.04) and decreased in 3 months of treatment (adj. *p* < 0.001). No association was found between longitudinal sGFAP levels and progressor status. sGFAP at baseline and 12 months was significantly associated with normalized ventricular (positively), thalamic (negatively), and lesion volumes (positively). Baseline and 12-month sGFAP predicted annualized ventricle volume change rate after 1 year of treatment. sGFAP correlated with sNfL at baseline (*p* < 0.001) and last sample follow-up (*p* < 0.001) but stabilized earlier.

**Discussion:**

sGFAP levels related to MRI markers of neuroinflammation and neurodegeneration.

## Introduction

High-efficacy treatment for relapsing-remitting multiple sclerosis (RRMS) has been successful in reducing relapses and radiological activity in most patients.^1–4^ One such high-efficacy treatment is natalizumab, which has been shown to substantially reduce relapses and radiological activity in most RRMS patients, particularly after 1 year of treatment.^1,2^ However, part of natalizumab-treated patients accumulate disability progression independent of disease activity (PIRA) despite highly effective treatment.^1,5,6^ We hypothesized true PIRA being caused by different neurodegenerative mechanisms as those behind the inflammatory activity.

Biomarkers explaining, predicting, or monitoring this progression are needed. Imaging biomarkers have previously been explored to explain the underlying mechanisms.^7–9^ One such biomarker, brain atrophy, could quantify magnetic resonance imaging (MRI)-derived neurodegeneration in treated cohorts.^7,8,10^ Blood-based biomarkers have also been proposed as a proxy to imaging or cerebrospinal fluid (CSF)-based biomarkers, offering an easily accessible and relatively non-invasively collected alternative.^11^ The identification of blood-based biomarkers in treated patients enabling explanation of the underlying neurodegenerative mechanisms is thus greatly relevant in consideration of explaining, predicting, or monitoring disease progression and possibly brain atrophy.

Glial fibrillary acidic protein (GFAP), a filament protein, plays a key role in the physiology of the cytoskeleton and consequently cell structure of the astrocyte.^12^ GFAP is mainly expressed in the central nervous system (CNS). This is in contrast to other biomarkers such as neurofilament light (NfL), which is also released in peripheral nerve damage.^13^ Due to these characteristics, serum GFAP (sGFAP) has been of interest in MS research. sGFAP has previously been shown to strongly correlate with CSF levels, supporting its use as a reliable blood-based biomarker.^14^ Additionally, astrocytes expressing GFAP are found in chronic MS plaques and previous studies have moreover shown a positive association between sGFAP, lesion load, and brain atrophy in RRMS and secondary progressive MS (SPMS).^15,16^ The exclusivity of GFAP to the CNS and the association of sGFAP with lesions and brain atrophy in RRMS theoretically make sGFAP an interesting blood-based biomarker to investigate in the context of disease progression in treated patients.

The aim of this study was to investigate sGFAP level dynamics in a natalizumab-treated RRMS cohort after treatment initiation. This offered an opportunity to investigate sGFAP during early treatment when neuroinflammation may still be present and during the neurodegenerative phase where the aforementioned inflammation has been largely suppressed. sGFAP levels during both phases were assessed for their relationship with clinical characteristics and ability to discern and predict disease progression (despite substantial suppression of focal inflammation) according to Expanded Disability Status Scale (EDSS)-plus criteria.^17^ In addition, we explored if sGFAP levels were associated with MRI volume measurements during both phases, as sGFAP was previously found to correlate with lesion load in MS and MRI volume measurements in both MS and other disease entities.^15,16,18–21^

We next tested if sGFAP levels could predict yearly MRI volume change rate, testing the ability of sGFAP to predict brain atrophy. We finally compared the results with those previously obtained for serum NfL (sNfL) in the same cohort. sNfL is another (widely investigated) proposed biomarker in MS and sGFAP correlated to sNfL levels in other cohorts but did not correlate with disability progression in this natalizumab-treated cohort.^15,22^

## Methods

### Participants

We selected 88 natalizumab-treated RRMS patients with a minimum follow-up of 3 years from a previously described ongoing observational cohort study.^23^ Baseline was defined as the first natalizumab infusion. The last clinical follow-up was defined as either the last visit before natalizumab discontinuation or the last visit before final data collection in November 2020. Yearly clinical assessments were done at baseline and every year thenceforth and included an EDSS, nine-hole peg test (9HPT), and timed 25-foot walk test (T25FW). Patients were divided into progressors and non-progressors based on significant disability progression between year 1 and the last follow-up, using yearly clinical assessments. Year 1 was chosen to correct for disability due to residual inflammation or anti-inflammatory disease improvement after natalizumab initiation. EDSS, 9HPT, and T25FW assessments within 1 year of relapse were excluded. We aimed to measure PIRA in natalizumab-treated RRMS using these clinical measurements by defining it as significant disability progression, hereafter referred to as progressor status, as an increase of EDSS by 1.5, 1.0, or 0.5 points by a reference EDSS of, respectively 0, 1.0–5.0, or ⩾5.5, or a 20% change in 9HPT or T25FW.^17^

### Serum GFAP and NfL measurements

Samples were collected at baseline and every 3 months onward. Blood was centrifuged at 1800 *g* for 10 minutes at room temperature, aliquoted, and stored at −80°C. sGFAP was measured using the Simoa™ GFAP Discovery Kit on the Simoa™ HD-X instrument following the instructions (Quanterix, Billerica, USA). Intra- and inter-assay precision of three quality control samples measured in duplicate over four runs ranged from 3.0% to 8.3% and 3.4% to 6.3%. sNfL was measured by the Simoa NF-light® Advantage Kit on the Simoa™ HD-X instrument following the instructions (Quanterix, Billerica, USA). Intra- and inter-assay precision of three quality control samples measured in duplicate over four runs ranged from 2.6% to 4.3% and 4.9% to 11.9%.

### Magnetic resonance imaging and processing

MRI scans (including 2D T1- and PD/T2-weighted) were collected yearly after the initial baseline scan within 3 months of baseline. Radiological disease activity was defined as new/enlarged T2 hyperintense and/or, if available, T1 gadolinium-enhancing lesions. Assessments were performed by neuroradiologists.

MRI scans were obtained in clinical practice; hence, subjects were scanned on various MRI scanners over the years (Supplemental eTable 1). PD/T2-weighted MRI scans were found to be most consistently available in clinical protocols compared with T1-weighted scans. Brain volumes and volume changes were accordingly assessed on the PD/T2-weighted MRI scans, as previously described in this cohort.^10^ Longitudinal brain segmentation was performed with sequence adaptive multimodal segmentation (SAMSEG) method, a method validated on T2 as provided in the open-source package FreeSurfer 7.1.1.^24,25^ The longitudinal pipeline of the SAMSEG method is designed with the ability to handle MRI data from different sources, such as different scanners and sequences encountered in longitudinal clinical cohorts, by adapting to the different protocols and making use of the shared information across intrapersonal repeated scans.^26^ Brain tissue segmentation was performed, and brain structure volumes were calculated using previously described methods.^10^

Normalized whole brain volume, ventricular volume, thalamic volume, and lesion volume were defined as a fraction and divided by intracranial volume. To calculate annualized percentage whole brain volume change rate, subject-wise linear regression was applied to volume measurements performed between year 1 and last follow-up to calculate their slope of change. The same procedure was performed for annualized percentage ventricular, thalamic, and lesion volume change rates.^10^ Measurements between baseline and year 1 were excluded for the calculation of annualized percentage change rate and linear mixed-effects analyses of volume measurements to rule out the effects of pseudo-atrophy.^27,28^

### Statistical analyses

Statistical analyses and visualizations were performed with R statistical software version 4.0.3.

Clinical and radiological characteristics were compared between groups using the Chi-square test for categorical variables and the *t*-test for normally and the Mann–Whitney U test for non-normally distributed continuous variables.

sGFAP and sNfL level differences between time points (baseline, 3(3M), 12(12M), and 24 months (24M) after initiation, and last sample follow-up) were compared using the Wilcoxon signed-rank test with post hoc Bonferroni adjustment. sGFAP levels at the time points were cross-sectionally compared between progressor groups using the Mann–Whitney U test.

Linear regression analyses were performed to test the cross-sectional association of sGFAP and sNfL levels with MRI volume measurements at baseline and 12M. Longitudinal sGFAP association with progressor status and brain volume measurements after 1 year of treatment was investigated using linear mixed-effect modeling (to account for pseudo-atrophy^27,28^). Due to non-normal distribution, sGFAP and sNfL were natural log transformed for mixed-effect and regression modeling. Disease duration at baseline was square-root transformed. Correction for gender, age, disease duration, clinical follow-up duration, and presence of gadolinium enhancement was performed in regression and mixed-effects analyses. Post hoc Holm–Bonferroni adjustment for multiple comparison correction was performed in linear regression analyses.

The predictive value of sGFAP at baseline and 12M for progressor status and the annualized percentage volume changes between 1 year of natalizumab treatment and follow-up were tested using logistic and linear regression analyses, respectively. Post hoc Holm–Bonferroni adjustment for multiple comparison correction was performed in linear regression analyses.

Finally, forward step selection (*p* value of <0.05) was performed using sGFAP levels at time points, sNfL levels at time points, and clinical and radiological characteristics to determine the best possible prediction model for whole brain, ventricular, and thalamic volume change rates. Correction for sex and age at baseline was performed on the predictive models.

A *p* value of <0.05 was considered significant.

### Ethical considerations

The Institutional Review Board (Medical and Biobank Ethics Committee of Amsterdam UMC, location VUmc) approved the use of routine medical files for research purposes (registration no. 2016.554). All subjects gave written informed consent for the collection and use of medical data and biological fluids for research purposes. This study adhered to the ethical principles of the Declaration of Helsinki.

## Results

### Comparing cohort characteristics

The median follow-up duration was 7.15 years. A total of 47.7% of the included patients showed significant disability progression. Of the 84.7% experiencing a relapse in the year before therapy initiation, the average time between relapse and initiation was 4.6 months (±3.1). The average months to previous relapse in progressors were 5.0 (±3.7) and 4.2 months (±2.5) in non-progressors. Clinical and radiological characteristics were compared between progression groups (Table 1). Baseline characteristics, normalized MRI volume measurements, and annualized percentage changes showed no difference between progression groups.

**Table 1.** Baseline and follow-up clinical and radiological characteristics and serum GFAP levels.

	Non-progressor (*N* = 46)	Progressor (*N* = 42)	Total (*N* = 88)	*p* Value
Female (%)	76.1	73.8	75.0	NS
Age (years)	35.6 ± 8.6	38.2 ± 8.4	36.9 ± 8.6	NS
Disease duration at baseline (years)	7.36 (3.83–11.8)	7.61 (4.17–12.7)	7.36 (3.83–12.1)	NS
Duration of clinical follow-up (years)	7.08 (4.47–9.69)	8.34 (5.78–11.2)	7.15 (4.92–10.3)	.038
Duration of sample follow-up (years)	4.75 (4.00–6.08)	5.55 (5.00–7.06)	5.17 (4.31–6.69)	.0075
Patients with relapses (%)				
1 year prior to baseline	83.7	85.7	84.7	NS
During first year of NTZ	10.9	19.0	14.8	NS
After 1 year of NTZ	10.9	7.14	9.1	NS
Baseline MRI				
With T1 GE (%)	70.0	67.5	68.8	NS
T1 GE lesion load (amount of lesions)	2.50 (0–7.00)	1.00 (0–3.25)	2.00 (0–5.25)	NS
T2 load ⩽ 38 lesions (%)	65.1	65.9	65.5	NS
T2 lesion load if ⩽38 lesions (lesions)	25.0 (15.0–30.0)	29.5 (17.3–33.0)	26.0 (15.0–30.0)	NS
Radiological activity^a^ (%)				
During first year of NTZ	28.3%	32.5%	30.2%	NS
After 1 year of NTZ	6.52%	9.52%	7.95%	NS
Disability at baseline				
EDSS	3.5 (2.5–5.0)	4.0 (2.5–5.5)	3.5 (2.5–5.0)	NS
9-HPT (seconds)	20.7 (18.7–26.4)	22.7 (20.6–25.2)	21.6 (19.8–26.2)	NS
T25FW (seconds)	4.8 (3.7–6.1)	5.50 (4.3–8.28)	4.90 (3.9–7.3)	NS
Disability at 12M				
EDSS	3.0 (3.0–4.0)	4.0 (3.0–6.0)	4.0 (3.0–5.0)	NS
9-HPT (seconds)	20.7 (19.4–26.0)	22.8 (20.2–25.8)	21.7 (19.7–26.0)	NS
T25FW (seconds)	4.7 (3.5–5.7)	5.20 (4.3–7.7)	4.85 (4.0–6.1)	NS
Disability at last clinical follow-up				
EDSS	3.5 (2.0–4.0)	5.0 (3.5–6.0)	4.0 (3.0–5.5)	<0.001
9-HPT (seconds)	20.3 (18.6–25.3)	24.1 (21.2–30.1)	23.1 (19.9–27.9)	0.024
T25FW (seconds)	4.3 (3.8–5.8)	5.8 (4.8-10.4)	5.0 (4.1–7.4)	<0.001
Serum GFAP (pg/ml)				
Baseline	116.4 (83.8–151.0)	102.8 (82.3–142.1)	110.8 (82.3–147.7)	NS
3M	90.4 (66.9–110.4)	86.9 (65.6–110.9)	88.5 (66.6–110.7)	NS
12M	89.2 (72.7–116.7)	87.4 (71.8–112.1)	88.7 (71.8–113.0)	NS
24M	84.1 (62.5–111.1)	90.9 (77.7–113.0)	87.5 (65.0–112.5)	NS
Last sample follow-up	97.0 (74.7–121.5)	91.9 (77.5–111.7)	94.0 (74.8–119.8)	NS
Serum NfL (pg/ml)				
Baseline	16.6 (10.8–29.6)	14.0 (9.76–20.8)	15.0 (10.1–26.6)	NS
3M	11.2 (8.72–15.4)	11.4 (8.04–16.4)	11.2 (8.54–15.9)	NS
12M	7.44 (5.94–10.5)	8.20 (6.65–11.0)	8.09 (5.96–11.0)	NS
24M	8.05 (5.80–10.2)	7.67 (5.74–11.1)	7.87 (5.75–10.5)	NS
Last sample follow-up	7.86 (5.45–11.6)	9.56 (6.47–10.5)	8.83 (5.59–11.2)	NS
Baseline brain fraction^b^ (%)				
Whole brain	71.9 (69.5–73.8)	71.2 (69.6–73.7)	72 (69.6–73.7)	NS
Ventricle	2.8 (2.2–3.5)	2.9 (2.2–3.4)	2.9 (2.2–3.5)	NS
Thalamus	0.8 (0.7–0.9)	0.8 (0.7–0.8)	0.8 (0.7–0.8)	NS
Lesion	0.8 (0.3–1.3)	0.5 (0.1–1.2)	0.6 (0.2–1.3)	NS
Annualized volume change (%)				
Whole brain, 12M-last clinical follow-up	−0.24 (−0.81 to 0.49)	−0.29 (−0.94 to 0.31)	−0.27 (−0.82 to 0.35)	NS
Ventricle, 12M-last clinical follow-up	0.09 (−1.19 to 1.24)	0.38 (−0.60 to 1.47)	0.23 (−0.78 to 1.32)	NS
Thalamus, 12M-last clinical follow-up	−0.36 (−0.84 to 0.54)	−0.45 (−1.14 to 0.17)	−0.40 (−1.00 to 0.21)	NS
Lesion, 12M-last clinical follow-up	−2.07 (−7.19 to 5.84)	−0.91 (−4.67 to 3.69)	−1.97 (−5.25 to 4.38)	NS

Mean values are displayed with ± standard deviation. Median values are displayed with (interquartile range). *p* Values were calculated using chi-square test for categorical variables and *t*-test and Mann–Whitney U test for normally and non-normally distributed continuous variables, respectively. Progressor status was defined as significant change in EDSS+ status.

NS: non-significant; NTZ: natalizumab; MRI: magnetic resonance imaging; GE: gadolinium-enhancing; FU: follow-up; EDSS: expanded disability status scale; 9-HPT: nine-hole peg test; T25FW: timed 25-foot walk test; EDSS+: EDSS with 9-HPT and T25FW; GFAP: glial fibrillary acidic protein; NfL: neurofilament light; 3M: 3 months after baseline; 12M: 12 months after baseline; 24M: 24 months after baseline.

a MRI activity was defined as new/enlarged T2 lesions and/or T1 gadolinium-enhancing lesions.

b Volumes were defined as fraction and were calculated as the sum of tissue, divided by intracranial volume.

### sGFAP levels before and after natalizumab initiation

**Figure 1.**
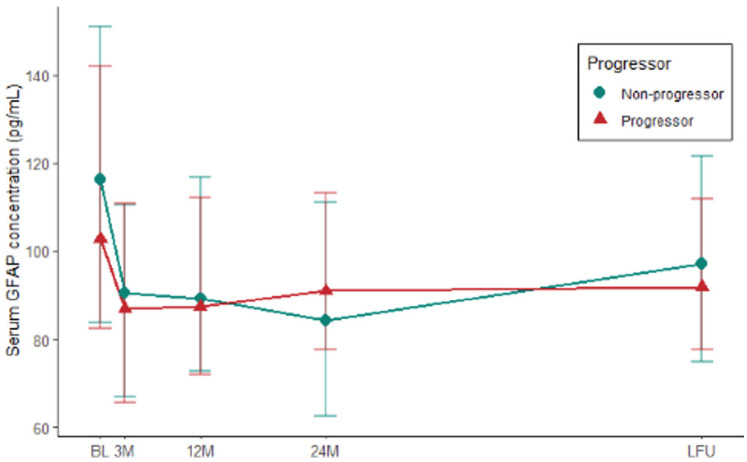
Longitudinal dynamics of serum GFAP in significant clinical progressors and non-progressors. A significant decrease of serum GFAP levels was seen in separate groups + groups combined. No significant difference was found between groups at each time point. Significant progress = based on expanded disability status scale with nine-hole peg test and timed 25-foot walk test (see Section “Methods”). GFAP: glial fibrillary acidic protein; BL: baseline; 3M: 3 months after baseline; 12M: 12 months after baseline; 24M: 24 months after baseline; LFU: last sample follow-up.

Median baseline sGFAP was significantly higher in patients with MRI gadolinium enhancement (117.00 vs. 88.16, 1.3 fold difference, *p* = 0.04). After natalizumab initiation, sGFAP levels decreased significantly between the first natalizumab infusion and 3 months of follow-up (effect size *r* = 0.64, adj. *p* < 0.001). This significant decrease was present in the entire cohort and was present in both progressors (effect size *r* = 0.59, adj. *p* < 0.001) and non-progressors (effect size *r* = 0.70, adj. *p* < 0.001). sGFAP levels did not change significantly after 3 months of treatment (Figure 1, Supplemental eFigure 1).

### sGFAP and progressor status

There was no significant difference in sGFAP levels between progressors (median values during follow-up ranged 85.8–101.9 pg/ml) and non-progressors (median values during follow-up ranged 84.1–116.4 pg/ml) at all time points (BL: *p* = 0.49, M3: *p* = 0.71, M12: *p* = 0.74, M24: *p* = 0.53, and LFU: *p* = 0.83; see Table 1 for levels at each time point). This remained when excluding patients experiencing relapses or radiological activity after 1 year of treatment. Baseline sGFAP levels did not predict progressor status in binary logistic regression analysis (OR = 0.996, *p* = 0.32), M12 sGFAP neither predicted progressor status (OR = 0.996, *p* = 0.54). This remained unchanged after correction for age, gender, disease duration, and EDSS (at the respective time point). The generalized linear mixed-effects model showed no significant association of sGFAP levels over time with progressor status when accounting for in-between subject variation. This result remained unchanged when correcting for age, gender, and disease duration at baseline.

### sGFAP and subscores

Subscores (EDSS, 9HPT, and 25FWT) were assessed separately for association with sGFAP levels at baseline (Supplemental eFigure 1). A significant association was found between sGFAP and 9HPT at baseline (*p* = 0.007) and sGFAP and EDSS at LFU (*p* = 0.027). This changed when correcting for age, gender, disease duration, and BMI: no significant association was found between sGFAP and subscores at all time points.

### sGFAP and MRI volume measurements

**Figure 2.**
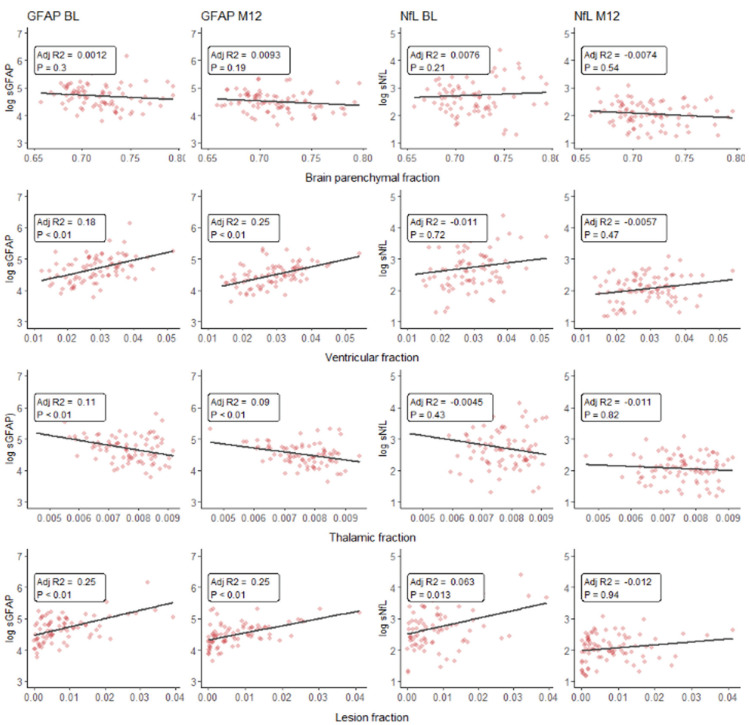
Scatterplots and univariate linear regression (uncorrected and unadjusted) line plot of natural log transformed sGFAP and sNfL levels versus normalized MRI volume measurements (expressed as fraction of intracranial volume) at baseline and 1 year of treatment. sGFAP: serum glial fibrillary acidic protein; sNfL: serum neurofilament light; BL: baseline; 3M: 3 months after baseline; 12M: 12 months after baseline.

sGFAP significantly predicted normalized lesion volume at baseline (std. β = 0.49, adj. *p* < 0.01) and 12M (std. β = 0.40, adj. *p* < 0.01). sGFAP additionally significantly predicted normalized ventricular volume at baseline (std. β = 0.42, adj. *p* < 0.01) and 12M (std. β = 0.42, adj. *p* < 0.01) and significantly predicted thalamic volume at baseline (std. β = −0.35, adj. *p* = 0.01) and 12M (std. β =−0.28, adj. *p* = 0.04). Associations at baseline were corrected for age, sex, disease duration, and presence of gadolinium enhancement. Associations at 12M were corrected for age, sex, and disease duration. Univariate regression plots can be found in Figure 2.

When accounting for in-between subject variation and correcting for age, gender, and disease duration, linear mixed-effects models (Table 2) showed that, after 1 year of treatment, a higher level of sGFAP over time was associated with a higher normalized lesion volume (std. β = 0.09, *p* = 0.02) but was not associated with normalized whole brain (std. β = −0.05, *p* = 0.45), ventricular (std. β = −0.04, *p* = 0.16) or thalamic volumes (std. β = −0.02, *p* = 0.66).

**Table 2.** Linear mixed-effects models and parameter estimates for whole brain fraction, ventricle fraction, thalamus fraction, lesion fraction, and a second lesion fraction model (with exclusively year 1 GFAP values).

Whole brain fraction			Ventricular fraction		
AIC = 553.2			AIC = 215.2		
Fixed effects	Std. β	*p* Value	Fixed effects	Std. β	*p* Value
Baseline age	−0.08 (−0.28 to 0.11)	0.402	Baseline age	0.1 (−0.13 to 0.34)	0.373
Female sex	0.17 (−0.23 to 0.57)	0.411	Female sex	−0.13 (−0.6 to 0.35)	0.601
Disease duration	−0.27 (−0.47 to −0.07)	0.008	Disease duration	0.23 (−0.01 to 0.46)	0.059
Ln GFAP	−0.05 (−0.17 to 0.08)	0.447	Ln GFAP	−0.04 (−0.09 to 0.01)	0.162
Time	−0.21 (−0.28 to −0.14)	<0.001	Time	0.03 (0.01–0.06)	0.002
Ln GFAP × time	−0.03 (−0.09 to 0.04)	0.441	Ln GFAP × time	0.01 (0–0.04)	0.148
Thalamic fraction			Lesion fraction		
AIC = 330.1			AIC = 323.4		
Fixed effects	Std. β	*p* Value	Fixed effects	Std. β	*p* Value
Baseline age	0.14 (−0.09 to 0.36)	0.227	Baseline age	0.01 (−0.2 to 0.23)	0.887
Female sex	0.25 (−0.2 to 0.7)	0.275	Female sex	0.26 (−0.18 to 0.7)	0.243
Disease duration	−0.35 (−0.57 to −0.12)	0.003	Disease duration	0.26 (0.05–0.48)	0.018
Ln GFAP	−0.02 (−0.09 to 0.06)	0.659	Ln GFAP	0.09 (0.01–0.16)	0.019
Time	−0.09 (−0.13 to −0.06)	<0.001	Time	−0.06 (−0.09 to −0.03)	<0.001
Ln GFAP × time	0.01 (−0.02 to 0.04)	0.657	Ln GFAP × time	−0.07 (−0.1 to −0.04)	<0.001

For GFAP levels and MRI volumes, time points included were 12M, 24M, and last clinical follow-up. GFAP levels were natural log transformed.

AIC: Akaike information criteria; sGFAP: serum glial fibrillary acidic protein; 12M: 12 months after baseline; 24M: 24 months after baseline.

Regression analysis furthermore showed that baseline sGFAP significantly predicted annualized ventricle volume change rate after correcting for age, gender, disease duration, clinical follow-up duration, and presence of gadolinium enhancement at baseline (std. β = 0.32, adj. *p* = 0.02). After correction for age, gender, disease duration, and clinical follow-up duration, sGFAP levels at 12M significantly predicted annualized ventricular change rate (std. β = 0.32, adj. *p* = 0.02).

### Comparison with results obtained for sNfL

**Figure 3.**
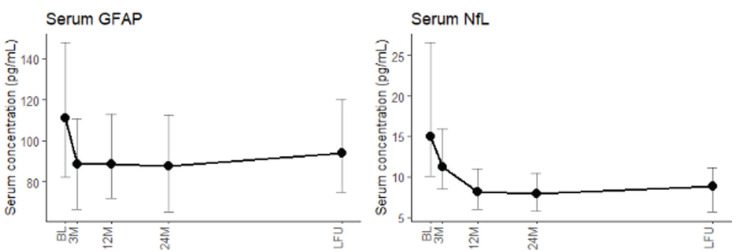
Longitudinal dynamics of serum GFAP levels and serum NfL levels. A significant decrease in serum GFAP levels was seen in the first 3 months, and a significant decrease of serum NfL levels was seen in the first 3 months (with its nadir at 1 year of treatment).^22^ Serum GFAP and NfL correlated at baseline and last follow-up. GFAP: glial fibrillary acidic protein; NfL: neurofilament light; BL: baseline; 3M: 3 months after baseline; 12M: 12 months after baseline; 24M: 24 months after baseline; LFU: last sample follow-up.

sNfL level dynamics during sample follow-up were compared with sGFAP level dynamics during sample follow-up (Figure 3). Akin to sGFAP, sNfL decreased significantly in the first 3 months of treatment (effect size *r* = 0.65, *p* = 0.001). However, sGFAP stabilized after 3 months of treatment, while sNfL continued to decrease between 3 months and 1 year of treatment (effect size *r* = 0.74, *p* < 0.001). sGFAP correlated significantly with sNfL levels at baseline (*p* < 0.001) and last sample follow-up (*p* < 0.001), but not at 3M, 12M, and 24M. sNfL levels were significantly higher in patients with gadolinium enhancement at baseline (adj. *p* < 0.001). sNfL was not associated with normalized whole brain, ventricular, thalamic, or lesion volume at baseline or 12M after correction and post hoc adjustment for multiple comparisons. Finally, predictive modeling showed that sNfL levels at 12M were predictive of whole brain, ventricular, and thalamic volume change, whereas sGFAP levels were only predictive of ventricular growth (Table 3).

**Table 3.** Best prediction models for annualized change rates including both sGFAP and sNfL.

Annualized whole brain volume change rate Adj. *R*^2^ = 0.12, *F* = 3.93, *p* = 0.006		
Fixed effects	Std. β	*p* Value
Baseline age	−0.12	0.25
Female sex	0.11	0.29
Ln NfL at 12M	−0.27	**0.01**
Normalized WBF	−0.28	**<0.01**
Annualized ventricular volume change rateAdj. *R*^2^ = 0.18, *F* = 5.61, *p* = 0.006		
Fixed effects	Std. β	*p* Value
Baseline age	0.05	0.64
Female sex	−0.16	0.10
Ln NfL at 12M	0.26	**0.02**
Ln GFAP at 3M	0.79	**<0.01**
Annualized thalamic volume change rateAdj. *R*^2^ = 0.07, *F* = 3.00, *p* = 0.04		
Fixed effects	Std. β	*p* Value
Baseline age	−0.09	0.42
Female sex	0.16	0.14
Ln NfL at 12M	−0.25	**0.03**

Multivariate linear regression with forward selection procedure (cutoff *p* value **<** 0.05) was used to establish the best prediction model for the different MRI volume changes. sGFAP and sNfL levels were natural log transformed. Singificant *p*-values have been emboldened. sGFAP: serum glial fibrillary acidic protein; sNfL: serum neurofilament light; Ln: natural log transformed; 3M: 3 months after baseline; 12M: 12 months after baseline; WBF: whole brain parenchymal fraction of intracranial volume.

## Discussion

In our natalizumab-treated cohort, we investigated sGFAP dynamics in two phases: the early treatment phase and chronic, neurodegenerative phase. At baseline, before treatment started, we found higher sGFAP levels in the presence of gadolinium enhancement. sGFAP levels lowered significantly for 3 months after natalizumab initiation and stabilized afterward. In this natalizumab-treated cohort, sGFAP levels were not associated with significant disability progression during follow-up, as levels did not differ between significant disability progressors and non-progressors. Baseline sGFAP levels and 12M sGFAP levels did not predict disability progression during follow-up. However, sGFAP significantly predicted normalized lesion (positive), ventricular (positive), and thalamic volume (negative) during the first year of treatment. After 1 year of treatment, mixed-effects modeling showed longitudinal sGFAP levels remained significantly associated with normalized lesion volume. Moreover, sGFAP at baseline and 12M predicted annualized ventricular volume change rate in this cohort. Finally, sGFAP decreased quicker compared with sNfL but did correlate with sNfL levels at baseline and last follow-up. sNfL levels were also higher in presence of gadolinium enhancement and significantly predicted lesion volume at baseline and 12M of treatment.

Our results are in line with a previous study that showed sGFAP levels decreasing under varying, but unstratified, disease modifying treatment.^16^ To the best of our knowledge, this is the first study that shows the dynamics of sGFAP under a single high efficacy treatment or second-line therapy (natalizumab) in RRMS and compared it with sNfL dynamics. sGFAP dynamics in our natalizumab-treated cohort imply value for sGFAP as a quicker alternative treatment response biomarker when compared with sNfL, as sGFAP reached its nadir in 3 versus 12 months for sNfL.

Previous work reported conflicting results regarding the relationship of sGFAP with disability progression in RRMS but suggested a relationship between sGFAP and atrophy or lesion load MRI measurements.^14–16,29,30^ We provided no evidence for a connection between sGFAP and clinical outcomes. In contrast, we found a relationship between sGFAP and MRI measurements. The relationship between sGFAP and lesion load in our cohort is supported by the results showing sGFAP relating to inflammation and lesion development as seen at baseline. The association between sGFAP and lesion volume was furthermore present during the entire follow-up. This could indicate that astrocyte activity remains involved in MS lesions, independent of suppressing the transmission of inflammatory cells into the CNS.

Interestingly, we found evidence that sGFAP and thus astrocytes are involved in not only active inflammation but also chronic processes such as brain atrophy, as sGFAP levels predicted ventricular enlargement. Ventricular enlargement is more prominent in RRMS compared with progressive MS and was linked to disability.^31,32^ A biomarker that could effectively monitor or even predict this enlargement in RRMS could be of great value.

This study has potential limitations. We chose a robust clinical outcome, which helped us identify significant disability progression in our relatively smaller sample size but complicated finding of subtle disability progression. The smaller sample size made the MRI measurements more susceptible to variation in results due to the different MRI scanners used for each individual during the relatively long clinical follow-up. We sought to correct for this using the SAMSEG-method, which aims to adapt to different scanners and sequences.^24,26^ Finally, while recent publications have shown body mass to influence sNfL, this was unknown at the time of therapy initiation and, as such, no body mass indexes were available.^33,34^

To conclude, we demonstrate that levels of sGFAP are related to both acute and chronic MRI measurements: suggesting a role of astrocytes in both acute and chronic phases of RRMS. In the acute phase, sGFAP related to inflammation and showed promise as alternative for monitoring treatment response to sNfL.^12,13^ In the chronic phase, compared with sNfL, sGFAP is more strongly related to cross-sectional MRI measurements. sGFAP related to brain atrophy measurements as well, albeit less so compared with sNfL (which showed more predictive value). Importantly, sGFAP levels failed to capture long-term clinical progress in our cohort, in both composite and separate clinical scores. We therefore suggest a role for sGFAP as tool for inflammation, treatment response, and radiological progression, but do not provide evidence supporting its use as biomarker for predicting and monitoring clinical progression in RRMS. Further research into sGFAP as an alternative biomarker to sNfL for inflammation and treatment response will likely yield clinically relevant results. Research into sGFAP and chronic radiological progression can expand knowledge on processes involved in neurodegeneration and brain atrophy in RRMS.

## Supplemental Material

sj-docx-1-msj-10.1177_13524585231188625 – Supplemental material for Serum glial fibrillary acidic protein in natalizumab-treated relapsing-remitting multiple sclerosis: An alternative to neurofilament light Click here for additional data file. Supplemental material, sj-docx-1-msj-10.1177_13524585231188625 for Serum glial fibrillary acidic protein in natalizumab-treated relapsing-remitting multiple sclerosis: An alternative to neurofilament light by Mark HJ Wessels, Zoë YGJ Van Lierop, Samantha Noteboom, Eva MM Strijbis, Johannes A Heijst, Zoé LE Van Kempen, Bastiaan Moraal, Frederik Barkhof, Bernard MJ Uitdehaag, Menno M Schoonheim, Joep Killestein and Charlotte E Teunissen in Multiple Sclerosis Journal

sj-docx-2-msj-10.1177_13524585231188625 – Supplemental material for Serum glial fibrillary acidic protein in natalizumab-treated relapsing-remitting multiple sclerosis: An alternative to neurofilament light Click here for additional data file. Supplemental material, sj-docx-2-msj-10.1177_13524585231188625 for Serum glial fibrillary acidic protein in natalizumab-treated relapsing-remitting multiple sclerosis: An alternative to neurofilament light by Mark HJ Wessels, Zoë YGJ Van Lierop, Samantha Noteboom, Eva MM Strijbis, Johannes A Heijst, Zoé LE Van Kempen, Bastiaan Moraal, Frederik Barkhof, Bernard MJ Uitdehaag, Menno M Schoonheim, Joep Killestein and Charlotte E Teunissen in Multiple Sclerosis Journal
