# Supplementary material for: Serum glial fibrillary acidic protein in natalizumab-treated relapsing-remitting multiple sclerosis: An alternative to neurofilament light
Source: Mult Scler. 2023 Aug 2;29(10):1229–39. doi: 10.1177/13524585231188625 (PMC10503252; doi:10.1177/13524585231188625)
Supplement: sj-docx-1-msj-10.1177_13524585231188625 – Supplemental material for Serum glial fibrillary acidic protein in natalizumab-treated relapsing-remitting multiple sclerosis: An alternative to neurofilament light [file sj-docx-1-msj-10.1177_13524585231188625.docx]

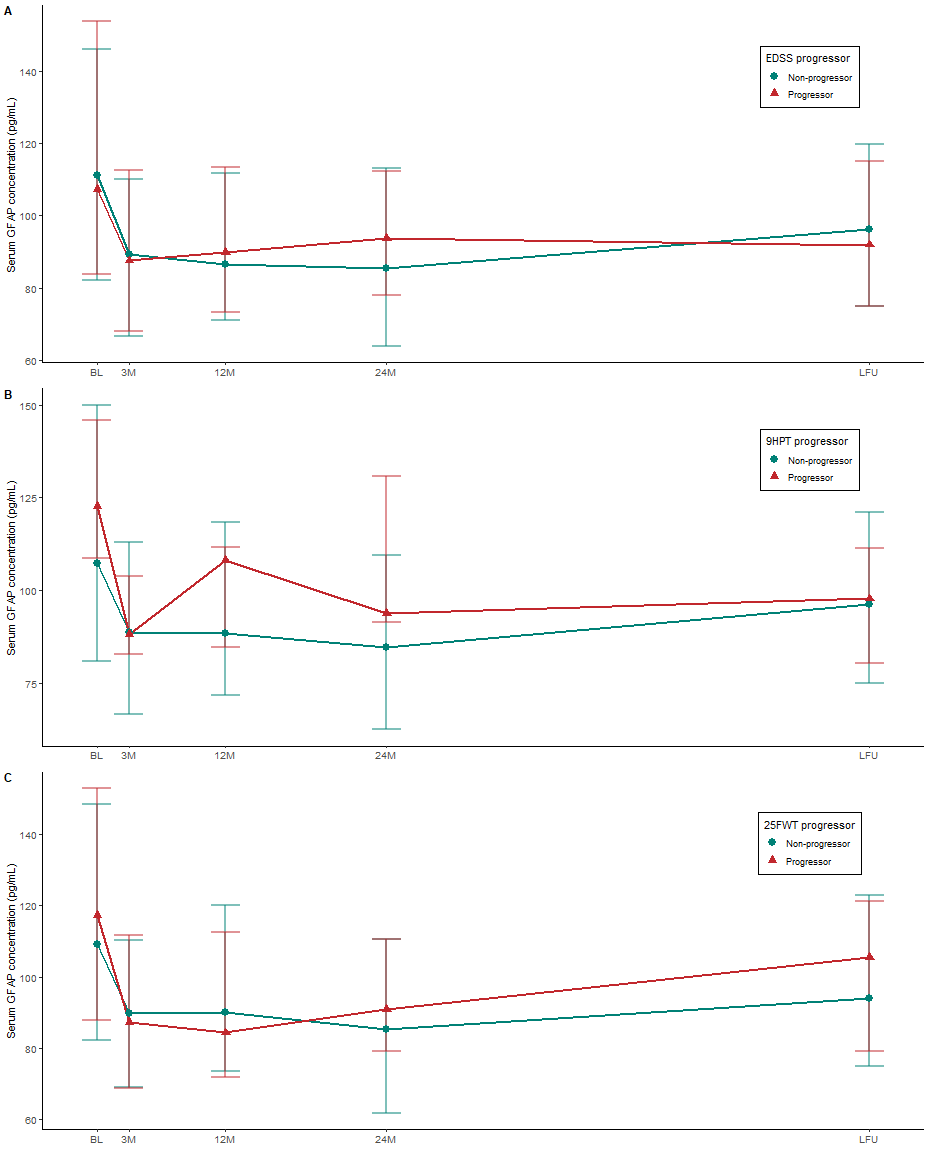


**eFigure 1**. Longitudinal dynamics of serum GFAP in significant progressors and non-progressors. No significant difference was found between groups at each time point.. A. Cohort divided based on significant progress in EDSS. B. Cohort divided based on significant progress in nine-hole peg test. C. Cohort divided based on significant progress in timed 25 foot walk test.

Significant progress = an increase of EDSS by 1.5, 1.0, or 0.5 points by a reference EDSS of respectively 0, 1.0-5.0 or ≥5.5, or a 20% change in 9HPT or T25FW. GFAP = glial fibrillary acidic protein. BL = baseline. 3M = 3 months after baseline. 12M = 12 months after baseline. 24M = 24 months after baseline. LFU = last sample follow-up.
