# Supplementary material for: Serum glial fibrillary acidic protein in natalizumab-treated relapsing-remitting multiple sclerosis: An alternative to neurofilament light
Source: Mult Scler. 2023 Aug 2;29(10):1229–39. doi: 10.1177/13524585231188625 (PMC10503252; doi:10.1177/13524585231188625)
Supplement: sj-docx-2-msj-10.1177_13524585231188625 – Supplemental material for Serum glial fibrillary acidic protein in natalizumab-treated relapsing-remitting multiple sclerosis: An alternative to neurofilament light [file sj-docx-2-msj-10.1177_13524585231188625.docx]

| ***MRI scanner*** | ***N=650 scans (%)*** |
| --- | --- |
| Siemens Sonata 1.5 T | 258 (36.7%) |
| GE Signa HDxt 1.5 T | 212 (32.6%) |
| Toshiba Titan 3.0 T | 62 (9.5%) |
| Siemens Avanto 1.5 T | 58 (8.9%) |
| Siemens Magnetom Vision 1.5 T | 19 (2.9%) |
| Siemens Magnetom Expert 1.5 T | 16 (2.5%) |
| GE Signa HDx 1.5 T | 13 (2.0%) |
| GE Discovery MR750 3.0 T | 5 (0.8%) |
| Philips Ingenuity 1.5 T | 3 (0.5%) |
| Philips Gyroscan 1.5 T | 2 (0.3%) |
| GE Signa Excite 3.0 T | 1 (0.2%) |
| Siemens Magnetom Espree 1.5 T | 1 (0.2%) |

**eTable1** Overview of MRI-scanners that were used during clinical follow-up of 88 patients.
